# Costs and repeat rates associated with colonoscopy observed in medical claims for commercial and Medicare populations

**DOI:** 10.1186/1472-6963-14-92

**Published:** 2014-02-26

**Authors:** Bruce Pyenson, Charles Scammell, Jonah Broulette

**Affiliations:** 1 Milliman, Inc., One Pennsylvania Plaza, 38th floor, New York, NY 10119, USA

**Keywords:** Colonoscopy, Bowel preparation, Actuarial analysis, Colorectal cancer screening, Claims data

## Abstract

**Background:**

Colorectal cancer is among the leading causes of cancer and cancer-related mortality in the United States. The incidence and mortality associated with CRC can be reduced with preventive screening. Inadequate bowel preparation has been associated with missed adenomas and the need for repeat colonoscopies.

**Methods:**

Separate claims source databases were analyzed to determine the costs associated with colonoscopy in the commercial and Medicare populations. Observed repeat rates for colonoscopy within 4 years of initial screening were also examined.

**Results:**

Among the 6 most commonly used billing codes, the average allowed cost for an episode of colonoscopy in 2010 was $2,146 in the commercial population and $1,071 in the Medicare population, with average cost sharing of $334 and $275, respectively. The portion of colonoscopies associated with a biopsy or polyp removal exceeded 50% in the commercial and Medicare populations. Approximately 57% of colonoscopies in the commercial population were associated with claims for a prescription bowel preparation product within 30 days prior to the procedure. Three branded and three generic bowel cleansing products accounted for approximately 75% of the total number of prescription claims for colonoscopy. Given literature reports that up to 25% of patients receive inadequate bowel preparation, the rate of repeat colonoscopy within 4 years of initial screening was lower than expected among patients who were not coded with common clinical reasons for early repeat: benign neoplasm, lesion, or polyp removed at initial screening colonoscopy.

**Conclusions:**

The reported rates of inadequate bowel preparation are 15% to 25%, but the rates of repeat colonoscopy found in our analysis are much lower; this is a risk concern considering the reported, significant miss rate of adenomas secondary to inadequate bowel preparation.

## Background

Colorectal cancer (CRC) is the fourth leading cause of cancer and the second leading cause of cancer deaths after lung cancer in the United States [1]. The lifetime risk of developing CRC is approximately 1 in 20 for both males and females [1]. CRC develops slowly over a period of several years [2], allowing an opportunity for screening and prevention.

CRC is one of the most preventable cancers, which helps explain why CRC screening has become a public health priority in recent years. Several groups, including the US Multi-Society Task Force on CRC, American College of Gastroenterology, US Preventive Services Task Force, and National Comprehensive Cancer Network, have published recommendations for CRC screening [3-6]. A patient’s screening strategy will depend on the individual’s risk, age, family history of CRC, and incidence of other serious gastrointestinal conditions. However, starting at 50 years of age, an individual of average risk for CRC should begin regular screening for CRC. Screening may include annual fecal occult blood tests or fecal immunochemical tests (with colonoscopy reserved for patients testing positive for CRC) or endoscopic procedures performed every 5 years for flexible sigmoidoscopy or every 10 years for colonoscopy. In some patients, more frequent screening or the use of a stool DNA test or computed tomography colonoscopy may be appropriate.

Screening of any type considerably reduces the mortality associated with the disease [7]. In average-risk patients, screening with colonoscopy has been demonstrated to decrease the incidence of CRC by 67% and CRC deaths by 65% [8]. Furthermore, colonoscopic screening and removal of adenomatous polyps reduced the mortality from CRC by 53% relative to the incidence-based mortality from CRC in the general population [9].

Screening for CRC remains underused compared with breast and cervical cancer screening. Data from the 2010 National Health Interview survey indicated that compliance with recommended screening for breast and cervical cancer exceeded 72% [10]. However, among adults 50 years and older 58.6% are current with recommended CRC screening according to data from the recent National Health Interview or 65.4% according to the telephonic Behavioral Risk Factor Surveillance surveys [10]. These estimates are below the 75% goal set by the American Cancer Society and the 70.5% goal set by the Department of Health and Human Services for adults ≥ 50 years [11,12].

Colonoscopy provides a visual diagnosis of inflamed tissues, abnormal growths, ulcers, and bleeding from the rectum and colon to the small intestine. It allows for both diagnostic assessment of the colon and certain therapeutic interventions (removal of polyps) during the same procedure. Poor bowel preparation deters procedural success and may lead to missed polyps and cancer [13], prolonged procedural time to clean the colon, and cancelled procedures [14]. Rates of poor preparation are commonly reported between about 15% and 25% [15-17], and can lead to a recommendation for repeat colonoscopy.

The objectives of this manuscript are to 1) analyze the average cost and cost sharing associated with screening colonoscopy, including the cost of products for bowel preparation, among the most commonly reported procedure codes in the commercial and Medicare populations, and 2) determine the repeat rate of colonoscopy in patients without evidence of high risk for CRC in the commercial and Medicare populations. We report the repeat rate separately for patients depending on whether the initial colonoscopy procedure included a biopsy or removal of polyp. We refer to the repeat rate for patients with an initial colonoscopy procedure not including a biopsy or removal of polyp as the "more restrictive" repeat rate. The repeat rate for all selected patients is referred to as the "less restrictive" repeat rate.

## Methods

Two claims source databases were used in this analysis; the commercial population data was derived from Truven MarketScan® and the Medicare population data was gathered from the Medicare 5% beneficiary sample. MarketScan includes inpatient, outpatient, and drug claims for individuals covered by benefit plans of large employers, health insurers, and government employers. Insured groups with high deductible health plans or capitation, and individuals >65 years or not associated with an active employee were excluded from the commercial population analysis. The Medicare 5% beneficiary sample is a limited data set that contains all Medicare Part A and Part B paid claims generated by a sample of Medicare beneficiaries. Exclusions in the Medicare population analysis included beneficiaries who were disabled, institutionalized, eligible for Medicaid, or enrolled in a health maintenance organization.

### Screening colonoscopy

To determine the cost of screening colonoscopy in the commercial and Medicare populations, colonoscopies performed in 2010 with a selected Healthcare Common Procedure Coding System (HCPCS) code were selected after exclusions for patients identified as having pre-exisitng high risk conditions based on International Classification of Disease (ICD-9) diagnosis codes. International Classification of Disease (ICD-9) diagnosis codes were analyzed (Table 1). All costs on the day of colonoscopy for outpatients were included. All facility costs for inpatients were excluded.

**Table 1:** Eligible codes to identify screening colonoscopies of interest

**Code**	**Definition**
*Healthcare Common Practice Coding System (HCPCS)*	
45378	Diagnostic/screening colonoscopy for non-Medicare patients
45380	Colonoscopy with biopsy single/multiple
45383	Colonoscopy with ablation of tumors, polyps, or other lesions not amenable to removal by hot biopsy forceps
45384	Removal of polyps or other lesions by hot biopsy
45385	Removal of polyps or other lesions by snare technique
G0121	Screening colonoscopy for other Medicare patients
*International Classification of Disease (ICD-9) Diagnosis for excluding patients with pre-existing high-risk conditions*	
V76.51	Special screening for malignant neoplasm, colon
211.3	Benign neoplasm of other parts of digestive system, colon

The average allowed and paid costs and patient cost sharing were analyzed in both populations. Claims for bowel preparation prescriptions, percentage of colonoscopies with prescriptions filled within 30 days prior to the date of screening colonoscopy, and the average cost of bowel preparation were evaluated in the commercial population.

The allowed amount for a service is a dollar amount typically considered payment-in-full for a member insured by an insurance company and its associated network of healthcare providers. The allowed amount is typically a discounted rate rather than the provider’s actual charge. The insurer directly pays the healthcare provider the paid amount. The patient bears responsibility for cost sharing, which may include copays, coinsurances, and deductibles.

### Repeat screening colonoscopy

To examine the repeat rate of screening colonoscopy in the commercial and Medicare populations, colonoscopies performed in 2005 with a repeat colonoscopy within 4 years (48 months in commercial population; 16 quarters in Medicare population) with an eligible HCPCS code and an eligible ICD-9 diagnosis code (Table 1) were analyzed. Colonoscopies beyond the first repeat screening were ignored. Patients without continuous enrollment throughout the study period were excluded. Some of the HCPCS codes could indicate a condition that would justify a follow-up before the usual 10 year period; these were colonoscopies whose code indicated a biopsy or removal of a benign neoplasm, lesion, or polyp occurred during the initial screening colonoscopy (HCPCS codes: 45378 and G0121; ICD-9 code: V76.51). The repeat rate for colonoscopy patients whose initial colonoscopy did not include one of these biopsy or polyp removal procedures was tabulated separately, which is characterized as the more restrictive repeat rate. The less restrictive repeat rate calculation includes all patients with an initial colonoscopy (HCPCS codes: 45378, 45380, 45383, 45384, 45385, or G0121; and ICD-9 codes: V76.51 or 211.3). The sample sizes for the less restrictive cohorts were 22,044 and 24,720 for commercial and Medicare, respectively. The sample sizes for the more restrictive cohorts were 8,133 and 5,858 for commercial and Medicare, respectively. Comparisons within the commercial and within the Medicare populations examined the percent of repeat screening colonoscopies using both the less restrictive and more restrictive repeat rate criteria.

## Results

### Screening colonoscopy

In the databases used, the total number of eligible screening colonoscopies performed in 2010 was 489,575 in the commercial population and 56,496 in the Medicare population. Data regarding costs associated with screening colonoscopy claims are summarized in Table 2 for the commercial population and Table 3 for the Medicare population. Among the procedure codes used for screening colonoscopies, the average allowed amount for commercial claims was about twice the amount allowed for Medicare claims ($2,146 vs $1,071). Average beneficiary cost sharing for screening colonoscopy was 15.6% in the commercial population and 25.7% in the Medicare population. Compared with the commercial population, the portion of claims in the Medicare population with a colonoscopy that included a code for biopsy or polyp removal was about 25% higher (51.6% vs 69.0%). The majority of claims (>85%) in the commercial and Medicare populations had no modifier codes and less than 1% of claims were associated with codes for discontinuation (53, 73, and 74) or the need to spend additional time cleaning the colon (22 and 52).

**Table 2:** Analysis of screening colonoscopies, commercial population

**Code**	**Screening colonoscopies,% N = 489,575**	**Average per colonoscopy**			
		**Allowed**	**Paid**	**Cost share**	**Coinsurance %**
		**$**	**$**	**$**	
All screening colonoscopies	100	2,146	1,812	334	15.6
Colonoscopies with no modifiers	88.4	2,096	1,780	316	15.1
*45378*	*31.9*	*1,769*	*1,546*	*222*	*12.6*
*45380*	*24.4*	*2,309*	*1,935*	*374*	*16.2*
*45383*	*1.6*	*2,568*	*2,127*	*440*	*17.2*
*45384*	*5.8*	*2,419*	*2,018*	*400*	*16.6*
*45385*	*19.8*	*2,332*	*1,945*	*386*	*16.6*
*G0121*	*4.8*	*1,664*	*1,454*	*210*	*12.6*
Colonoscopies with modifiers* 22, 52, 53, 73, and 74	0.4	1,911	1,650	260	13.6
Colonoscopies with other modifiers	11.2	2,548	2,071	477	18.7

* 22 = Service provided is greater than that usually required for the listed procedure.

52 = A service or procedure is partially reduced at the physician’s discretion.

53 = Termination of a surgical or diagnostic procedure due to extenuating circumstances or those that threaten the well-being of the patient.

73 = Discontinued outpatient hospital/ambulatory surgery center procedure prior to the administration of anesthesia.

74 = Discontinued outpatient hospital/ambulatory surgery center procedure after administration of anesthesia.

**Table 3:** Analysis of screening colonoscopies, medicare population

**Code**	**Screening colonoscopies,% N = 56,496**	**Average per colonoscopy**			
		**Allowed**	**Paid**	**Cost share**	**Coinsurance%**
		**$**	**$**	**$**	
All screening colonoscopies	100	1,071	795	275	25.7
Colonoscopies with no modifiers	86.3	1,073	795	278	25.9
*45378*	*4.8*	*836*	*606*	*229*	*27.5*
*45380*	*22.5*	*1,057*	*784*	*272*	*25.8*
*45383*	*2.6*	*1,240*	*917*	*323*	*26.1*
*45384*	*8.2*	*1,139*	*834*	*305*	*26.8*
*45385*	*35.7*	*1,196*	*889*	*307*	*25.7*
*G0121*	*12.4*	*762*	*564*	*198*	*26.0*
Colonoscopies with modifiers	0.8	849	623	225	26.6
22, 52, 53, 73, and 74 *					
Colonoscopies with other modifiers	13.0	1,067	808	258	24.2

* 22 = Service provided is greater than that usually required for the listed procedure.

52 = A service or procedure is partially reduced at the physician’s discretion.

53 = Termination of a surgical or diagnostic procedure due to extenuating circumstances or those that threaten the well-being of the patient.

73 = Discontinued outpatient hospital/ambulatory surgery center procedure prior to the administration of anesthesia.

74 = Discontinued outpatient hospital/ambulatory surgery center procedure after administration of anesthesia.

Over half of the people obtaining colonoscopies in the commercial analysis (56.6%) had a claim for a prescription bowel preparation within 30 days prior to the procedure. Approximately 100 different National Drug Codes were identified for products that could be for bowel preparation and nearly 60% of all bowel preparation claims were for a branded product. Six bowel preparation products accounted for approximately 75% of the total number of prescriptions; this included 3 branded and 3 generic products (Table 4). The average cost sharing for branded products was $29.62 and that of generic products was $8.31. The allowed or paid cost associated with bowel preparation products was under 1% of the average allowed or paid cost, respectively, of the colonoscopy in the commercial population.

**Table 4:** Common bowel preparation prescriptions filled within 30 days prior to screening colonoscopy, commercial population

		**Bowel preparation prescriptions,% N = 208,178**	**Average per prescription**		
**National Drug Code**			**Allowed**	**Paid**	**Cost share**
	**Product name**		**$**	**$**	**$**
Branded		42.1	16.83	8.52	8.31
*65649020175*	*Moviprep®*	*27.9*	*48.89*	*17.38*	*31.52*
*52268052101*	*HalfLytely® Bowel Preparation Kit*	*14.2*	*53.72*	*26.53*	*27.19*
*65649070141*	*Osmoprep®*	*6.0*	*66.40*	*31.65*	*34.76*
Generic		57.7	50.50	20.88	29.62
*10572040001*	*PEG-3350/NaCl/Na Bicarbonate*	*15.0*	*18.32*	*9.69*	*8.63*
*62175044601*	*PEG-3350/Electrolytes*	*6.1*	*10.43*	*3.00*	*7.43*
*68220013104*	*Trilyte*	*6.0*	*20.01*	*10.81*	*9.20*

Approximately 43% of screening colonoscopies in the commercial population did not have a claim for a prescription bowel preparation within 30 days prior to the procedure. Although patients may have filled the prescription earlier than the 30-day prior range examined, it appears as though there is significant use of over-the-counter bowel cleansing products that are paid by the patient. Popular over-the-counter products general cost less than $25.

### Repeat screening colonoscopy

**Figure 1:**
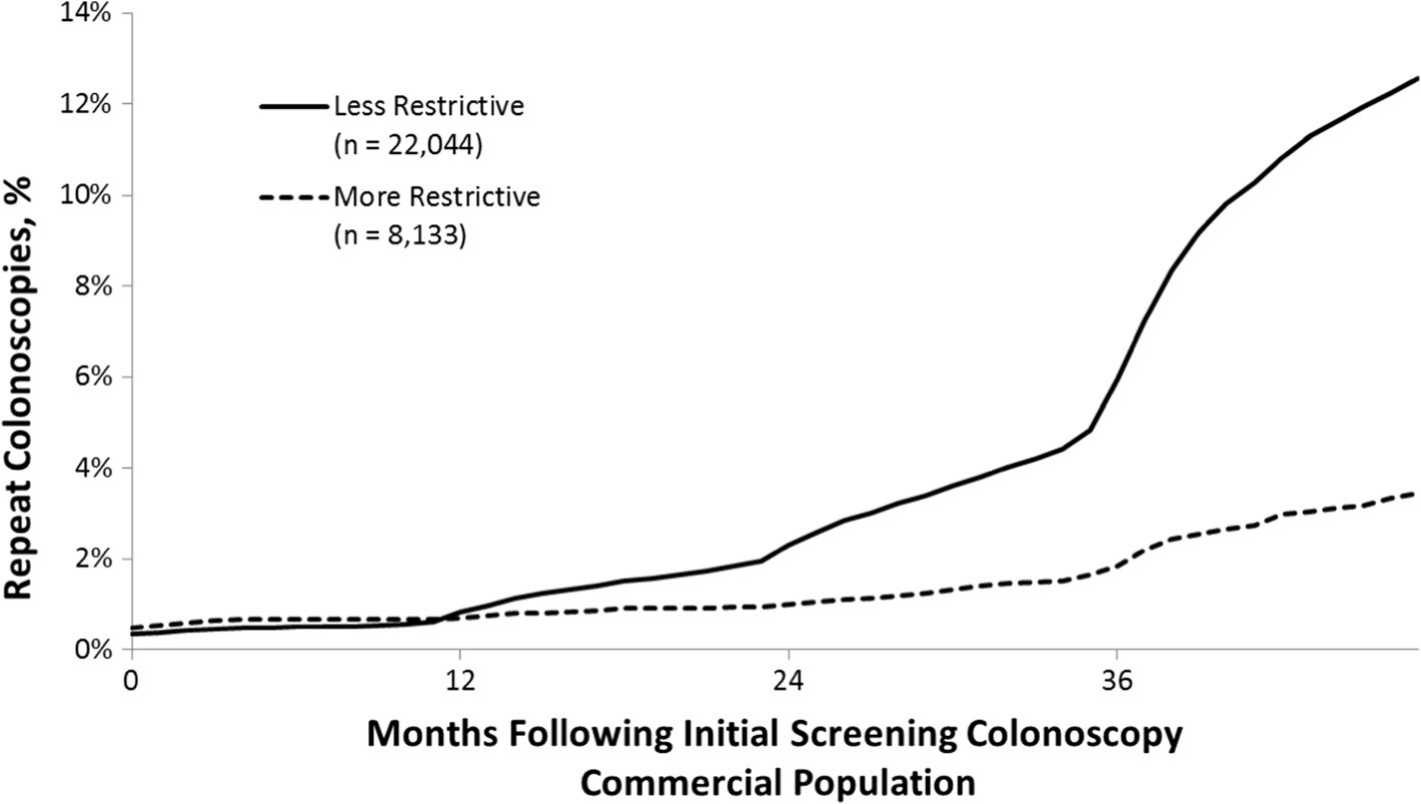
Cumulative colonoscopies repeated within 48 months following initial screening colonoscopy in the commercial population.

**Figure 2:**
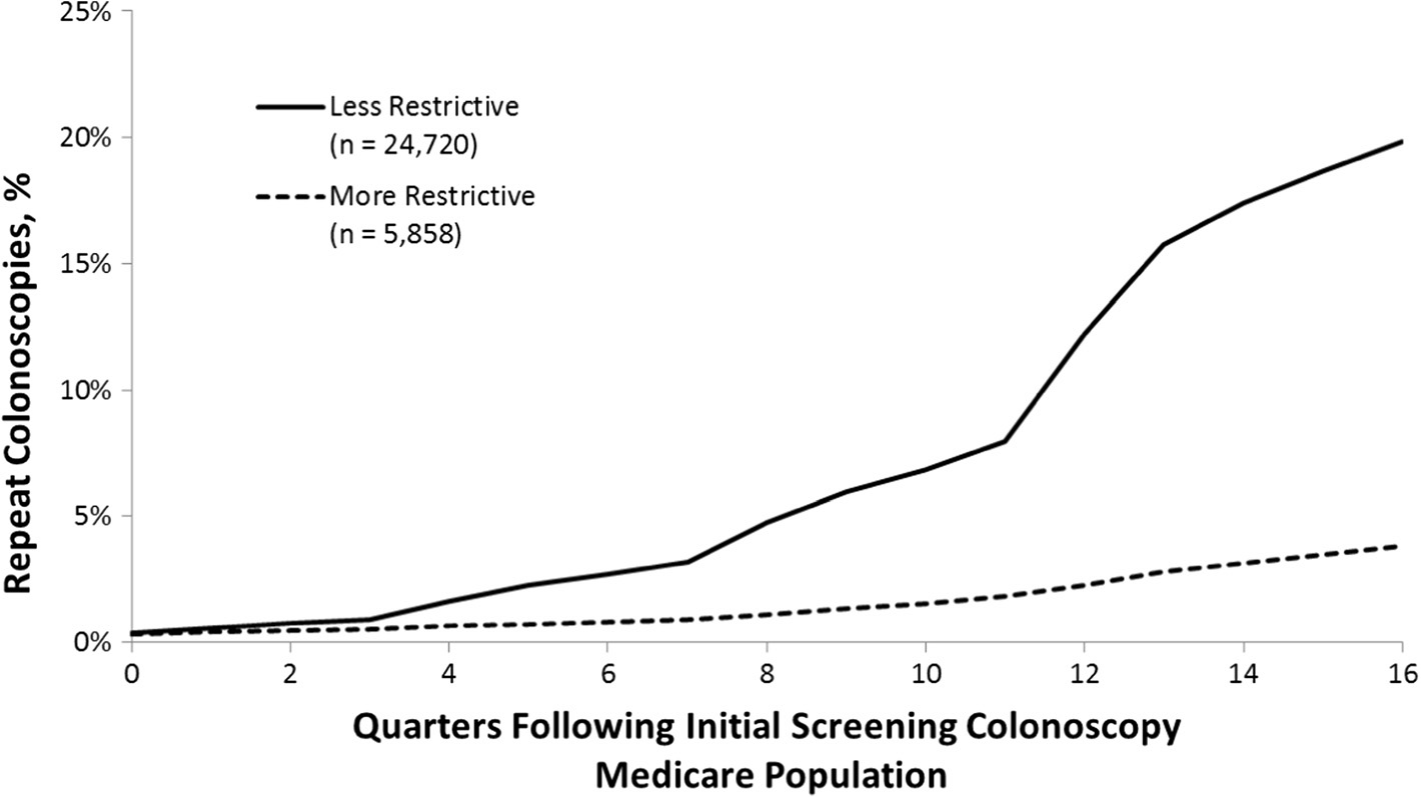
Cumulative colonoscopies repeated within 16 quarters following initial screening colonoscopy in the Medicare population.

Using the less restrictive repeat rate criteria, the portion of patients obtaining a repeat screening colonoscopy within 4 years of the initial screening was 12.6% and 19.8% for the commercial (n = 22,044) and Medicare (n = 24,720) cohorts, respectively. Using the more restrictive repeat rate criteria, the percentage of repeat screening colonoscopies within 4 years of the initial screening colonoscopy was 3.5% and 3.8% for the commercial (n = 8,133) and Medicare (n = 5,858) cohorts, respectively. See Figures 1 and 2.

## Discussion

Treating CRC is expensive; based on Medicare data from 2008 and 2009 we found the average cost is estimated at $43,000 within the first 12 months after diagnosis, not including prescription drug expense. Analysis of the MarketScan and Medicare 5% beneficiary sample claims databases indicated that the average allowed cost in 2010 for screening colonoscopy was $2,146 for commercial payers and $1,071 for Medicare patients. Colonoscopy has been established as a cost-effective screening technique because it reduces mortality at relatively low incremental costs on a population basis [18].

Our analysis revealed that more than 40% of colonoscopies did not have a claim for a prescription bowel preparation within 30 days prior to the procedure, suggesting the use of over-the-counter products in many patients.

Research indicates that approximately 1 in 4 to 1 in 6 patients are inadequately cleansed in preparation for colonoscopy [15-17]. Guidelines from the American Cancer Society and US Multi-Society Task Force on Colorectal Cancer recommend repeating the screening colonoscopy before planning a long-term surveillance program if the colon is not adequately prepared [19]. Given the high percentage of patients with inadequate preparation, the rate of repeat colonoscopy was anticipated to be higher than what was found in the commercial and Medicare populations. With suboptimal preparation, some clinicians may opt to recommend a shorter follow-up interval rather than repeat the colonoscopy [20], although our 4 year follow-up may include repeat colonoscopies as well as some with a shorter follow-up interval*.* The recent American College of Physician’s clinical guidance on colorectal cancer screening notes concern with over-use of colonoscopy as well as under-use, and the source databases are expected to include both [21]. The low rate of repeats found in our data is of concern as research indicates miss rate of adenomas secondary to inadequate bowel preparation is significant. Lebwohl reports 43% of adenomas were missed among asymptomatic patients undergoing repeat colonoscopy [16]; Chokshi reports 34% missed adenomas among average risk patients undergoing repeat colonoscopies, where repeats in both studies were due to inadequate preparation [13].

A limitation of this analysis is that the data did not capture the level of bowel preparation quality. In addition, the analysis relies on claims coding to make inferences, which can introduce biases such as caused by miscoding or under-coding. Also, the data from the analysis is based on historical numbers, and may not be appropriate for any one particular organization given the variability in health care benefits. However, the use of claims databases to identify individuals who have had screening colonoscopy seems supported by a recent comparison of medical records and claims [22].

Tabulating repeat rates only for lives having four years of continuous enrollment allowed for complete follow-up for all initial colonoscopies, but it could have introduced biases. For the commercial population, breaks in enrollment in the source database are typically due to shifts in employment or changing coverage such as obtaining Medicare coverage through aging or disability. Such individuals would have been excluded, and such individuals could be less healthy than those with continuous enrollment. For the Medicare population, breaks in enrollment are typically due to entry into Medicare Advantage or death. The impact of the continuous enrollment criteria on the results is unclear.

## Conclusions

The average allowed cost in 2010 for screening colonoscopy was $2,146 for commercial payers and $1,071 for Medicare patients. Given the reportedly high rates of patients with inadequate preparation for colonoscopy, the rate of repeat colonoscopy was anticipated to be higher than what was found in the commercial and Medicare populations. This is of risk concern because of significant miss rates of adenomas secondary to inadequate bowel preparation.

## Abbreviations

CRC: Colorectal cancer; HCPCS: Healthcare Common Procedure Coding System; ICD-9: International Classification of Disease.

## Competing interests

Financial support: Financial support for the analysis was provided by Ferring Pharmaceuticals Inc. The authors were not compensated for their work on this manuscript. Professional medical writing assistance was supported by Ferring Pharmaceuticals Inc.

Potential competing interests: The authors have no competing interest to declare.

## Authors’ contributions

CS, BP, and JP contributed to the study design, data analyses, and ensuing report of study results. BP, CS and JP helped to edit the manuscript. All authors read and approved the final manuscript.

## Pre-publication history

The pre-publication history for this paper can be accessed here:

http://www.biomedcentral.com/1472-6963/14/92/prepub
